# Therapeutic response of multidrug-resistant *Plasmodium falciparum* and *P. vivax* to chloroquine and sulfadoxine–pyrimethamine in southern Papua, Indonesia

**DOI:** 10.1016/j.trstmh.2006.06.008

**Published:** 2007-04

**Authors:** A. Ratcliff, H. Siswantoro, E. Kenangalem, M. Wuwung, A. Brockman, M.D. Edstein, F. Laihad, E.P. Ebsworth, N.M. Anstey, E. Tjitra, R.N. Price

**Affiliations:** a International Health Program, Menzies School of Health Research and Charles Darwin University, Darwin, NT, Australia; b National Institute of Health Research and Development, Jakarta, Indonesia; c MSHR-NIHRD Malaria Research Program, Timika, Papua, Indonesia; d Dinas Kesehatan Kabupaten, Timika, Papua, Indonesia; e Lembarga Pengembangan Masyarakat Amungme Kamoro, Timika, Papua, Indonesia; f Public Health & Malaria Control Department, PT Freeport Indonesia, Tembagapura, Papua, Indonesia; g Australian Army Malaria Institute, Brisbane, QLD, Australia; h Directorate General of Disease Control and Environment Health, Ministry of Health, Jakarta, Indonesia; i International SOS, Tembagapura, Papua, Indonesia; j Centre for Vaccinology & Tropical Medicine, Nuffield Department of Clinical Medicine, John Radcliffe Hospital, Oxford, UK

**Keywords:** Malaria, *Plasmodium falciparum*, *Plasmodium vivax*, Chloroquine, Sulfadoxine–pyrimethamine, Drug resistance, Papua, Indonesia

## Abstract

To determine the level of antimalarial drug resistance in southern Papua, Indonesia, we assessed the therapeutic efficacy of chloroquine plus sulfadoxine–pyrimethamine (CQ+SP) for *Plasmodium falciparum* infections as well as CQ monotherapy for *P. vivax* infections. Patients with *P. falciparum* failing therapy were re-treated with unsupervised quinine ± doxycycline therapy and those with *P. vivax* with either unsupervised quinine ± doxycycline or amodiaquine. In total, 143 patients were enrolled in the study (103 treated with CQ+SP and 40 with CQ). Early treatment failures occurred in four patients (4%) with *P. falciparum* and six patients (15%) with *P. vivax*. The failure rate by Day 28 for *P. vivax* was 65% (95% CI 49–81). After PCR correction for re-infections, the Day 42 recrudescence rate for *P. falciparum* infections was 48% (95% CI 31–65). Re-treatment with unsupervised quinine ± doxycycline resulted in further recurrence of malaria in 48% (95% CI 31–65) of *P. falciparum* infections and 70% (95% CI 37–100) of *P. vivax* infections. Eleven patients with recurrent *P. vivax* were re-treated with amodiaquine; there were no early or late treatment failures. In southern Papua, a high prevalence of drug resistance of *P. falciparum* and *P. vivax* exists both to first- and second-line therapies. Preliminary data indicate that amodiaquine retains superior efficacy compared with CQ for CQ-resistant *P. vivax*.

## 1 Introduction

The burden of malaria in Southeast Asia has been underappreciated by as much as 200% (Snow et al., 2005). Despite the efforts of the Roll Back Malaria initiative, the situation has deteriorated in the last 5 years as multidrug-resistant strains of plasmodia continue to spread across malaria-endemic communities.

Whereas chloroquine (CQ)-resistant *Plasmodium falciparum* in Indonesia was first documented over 30 years ago (Dondero et al., 1974), the emergence of drug-resistant strains of *P. vivax* was only described in 1989 in northern Papua Province (formerly Irian Jaya) on the eastern border of the archipelago (Baird et al., 1991). Within 5 years, the Day 28 failure rates following CQ therapy in this region had reached 95% for *P. falciparum* and 96% for *P. vivax* (Sumawinata et al., 2003). Other studies of *P. vivax* have subsequently confirmed a high prevalence of CQ resistance both in northern Papua (Baird et al., 1997a, Murphy et al., 1993, Taylor et al., 2001, Tjitra et al., 2002a) and elsewhere in Indonesia (Baird et al., 1996, Fryauff et al., 1998). High rates of resistance to second-line treatment (sulfadoxine–pyrimethamine (SP)) have been reported in Papua both for *P. falciparum* (Tjitra et al., 2001, Tjitra et al., 2002a) and for *P. vivax* (Tjitra et al., 2002b). The combination of CQ+SP has had similarly poor efficacy in northern Papua both against *P. falciparum* (Tjitra et al., 2002a) and *P. vivax* (Tjitra et al., 2002b).

Only one study has been published from southern Papua, which demonstrated that in 1992 52% of patients with *P. falciparum* had high-grade resistance (RII or RIII) to CQ monotherapy (Pribadi et al., 1998). In this study, in vitro sensitivity testing suggested that SP still retained efficacy against *P. falciparum*. Local policy in the Mimika district of southern Papua was changed to advocate CQ+SP for infections of *P. falciparum* and CQ monotherapy for *P. vivax*. However, in the ensuing decade a steady rise in the number of reported cases of malaria in this region despite a well-funded malaria control programme suggested the sustained emergence of significant antimalarial drug resistance. In a small unpublished study in 2001, failure rates by Day 28 of CQ+SP were 75% compared with 79% for SP alone (Crouch-Chivers, personal communication). The corresponding failure rates of CQ plus 3 days of primaquine resulted in a Day 28 cure rate of 13% for *P. vivax* infections.

As part of a series of studies to rationalise antimalarial protocols in this region, we undertook a series of chemotherapeutic trails to determine the efficacy of protocols for uncomplicated falciparum and vivax malaria that were prevailing in 2004.

## 2 Materials and methods

### 2.1 Study site

The study was carried out at an established rural outpatient clinic west of the city of Timika in southern Papua, Indonesia. This forested lowland area has unstable malaria transmission associated with three mosquito vectors: *Anopheles koliensis*, *A. farauti* and *A. punctulatus* (Lee et al., 1980, Pribadi et al., 1998). The annual incidence of malaria is 620 per 1000 per year, divided 55:45 between *P. falciparum* and *P. vivax* infections (Peter Ebsworth, personal communication). Owing to economic migration, the ethnic origin of the local population is diverse, with highland Papuans, lowland Papuans and non-Papuans all resident in the region. In view of the high number of infections in non-immune patients, local protocols recommend that all patients with patent parasitaemia are given antimalarial therapy.

### 2.2 Study design

This was a prospective, open-label drug efficacy study of protocols recommended in 2004 for infections with *P. falciparum* (CQ+SP) and *P. vivax* (CQ) in children and adults with uncomplicated symptomatic malaria. The study was based on the 2003 WHO in vivo antimalarial drug sensitivity protocol (WHO, 2003), modified to include mixed infections. Patients were followed for 42 days in the falciparum arm and 28 days in the vivax arm using a standardised drug efficacy record form.

### 2.3 Patients

Consecutive patients with slide-confirmed malaria (*P. falciparum*, *P. vivax* or mixed infections) with any parasitaemia and a fever or a history of fever during the preceding 48 h presenting to the outpatient clinic were enrolled in the study. Exclusion criteria included pregnant or lactating women, children under 10 kg, patients with signs of severity (WHO, 2000), a parasitaemia >4% or concomitant disease requiring hospital admission. Informed consent was signed by an adult patient or by a parent/guardian for children. If the subject was illiterate, consent was obtained in the presence of a literate witness. All patients were given an information sheet in their own language.

### 2.4 Study procedures

On admission, a standardised data sheet was completed recording demographic information, details of symptoms and their duration, and the history of previous antimalarial medication. Clinical examination findings were documented, including axillary temperature measured using a digital electronic thermometer. Venous blood was taken for blood film, haematocrit and white blood cell (WBC) count. Parasite counts were determined on Giemsa-stained thick films as the number of parasites per 200 WBCs. All slides were read by a certified microscopist with 10 years experience. A thick film was considered negative on initial review if no parasites were seen in 100 high power fields. A thin film was also examined to confirm parasite species and used for quantification if parasitaemia was >200 per 200 WBCs. All slides were cross-checked by another experienced microscopist.

Patients were examined daily thereafter until they became afebrile and aparasitaemic. At each visit, a blood film was taken and a symptom questionnaire was completed. Patients were then seen weekly for 4 weeks (*P. vivax* infections) or 6 weeks (*P. falciparum* or mixed infections). Patients were also encouraged to return to the clinic on any other day that they felt unwell. At each clinic visit a full physical examination was performed, the symptom questionnaire was completed and blood was taken to check for parasite count and haemoglobin level using a battery-operated portable photometer (HemoCue™ Hb201+; HemoCue, Angelholm, Sweden). Blood spots on filter paper (Whatman 3 mm chromatography paper) were also collected on Day 0 and on the day of failure.

### 2.5 Plasma chloroquine levels

Heparinised venous blood samples were taken from patients who agreed to be venipunctured on Days 0, 7, 28 or the day of recrudescence. Samples were processed within 4 h of venipuncture, spun at 2000 rpm for 10 min and plasma was removed and stored at −20 °C until analysis. Plasma drug concentrations of CQ and its major metabolite desethylchloroquine (DCQ) were assayed by HPLC as described previously (Alvan et al., 1982). The interassay coefficients of variation for CQ and DCQ were, respectively: 14.4% and 16.8% at 5 ng/ml (*n* = 6) and 2.5% and 5.3% at 100 ng/ml (*n* = 6). The limit of quantitation was 5 ng/ml for CQ and 2.5 ng/ml for DCQ. The minimum effective concentration (MEC) of CQ+DCQ was defined as 30 ng/ml plasma for *P. falciparum* and 15 ng/ml for *P. vivax* (Baird, 2004). These concentrations correspond to the whole blood concentrations used by other investigators of 200 ng/ml and 100 ng/ml, respectively (Baird et al., 1997b).

### 2.6 Treatment

Standard treatment courses of CQ+SP for *P. falciparum* (alone or mixed with *P. vivax*) or CQ for pure *P. vivax* infections were administered. The CQ+SP regimen consisted of CQ (P.T. Bayer, Jakarta, Indonesia; 150 mg base/tablet, 25 mg/kg over 3 days) and SP was given as a single dose on Day 0 (25 mg/kg sulfadoxine and 1.25 mg/kg pyrimethamine). All drug administrations were supervised and participants were observed for 30–60 min to exclude adverse reactions and to ensure the medication was not vomited. If vomiting occurred within 60 min, the whole dose was repeated once. If vomiting occurred again within 60 min, the patient was withdrawn from the study. Paracetamol was given if the axillary temperature was ≥38 °C.

Those patients failing therapy were re-treated with quinine (10 mg of salt/kg body weight/dosage orally three times a day for 7 days) plus doxycycline (100 mg twice a day for 7 days) if ≥8 years age and not pregnant. From May 2004, those patients with reappearance of *P. vivax* within 28 days were given a 3-day course of supervised amodiaquine if they consented (Flavoquine™; Aventis, Basel, Switzerland; 200 mg salt/150 mg base per tablet, 30 mg/kg over 3 days). Primaquine (15 mg base/kg of body weight for 14 days) was administered to those individuals with *P. vivax* infection or mixed infection on Day 28 of their participation in the study.

### 2.7 Endpoints

Therapeutic response was determined using clinical and parasitological criteria. The primary endpoints were the therapeutic response on the basis of parasitological and clinical cure by Day 28 for *P. vivax* infections and by Day 42 for *P. falciparum* infections. The WHO criteria (WHO, 2003) were modified to include *P. falciparum* genotyping to differentiate recrudescence from re-infection. The latter were distinguished according to polymorphisms in MSP-1, MSP-2 and GLURP, as described previously (Brockman et al., 1999). New infections detected by genotyping were classified as non-treatment failures. New infections and indeterminate PCR results were excluded from the Day 28 or Day 42 failure rate if the follow-up was terminated before the endpoint was achieved. Since CQ concentrations persist at levels above the MEC for *P. vivax* beyond 28 days (Baird et al., 1997b), any recurrence within this time was considered to be a therapeutic failure.

### 2.8 Statistical analysis

Data were double entered and validated using EpiData 3.02 software (EpiData Association, Odense, Denmark) and analysis was performed using SPSS for Windows (SPSS Inc., Chicago, IL, USA). The Mann–Whitney *U*-test or Kruskal–Wallis method were used for non-parametric comparisons, and Student's *t*-test or ANOVA were used for parametric comparisons. For categorical variables, percentages and corresponding 95% CI were calculated using Wilson's method. Proportions were examined using χ^2^ with Yates’ correction or by Fisher's exact test. In view of the frequent intercurrent infections with different species from the original, we also performed a survival analysis in which patients lost to follow-up or re-presenting with a different species from their original infection were censored on the last day of follow-up and regarded as not being treatment failures. Failure rates were derived from the cumulative incidence of failure at Day 28 or Day 42. Thresholds for defining an inadequate threshold for parasite reduction ratio (PRR) at 24 h (parasitaemia on day 1/parasitaemia on admission) were assessed using the receiver operator curve and Youden's Index calculated as sensitivity plus specificity minus 1.

### 2.9 Ethics

The study was approved by the Ethics Committee of the National Institute of Health Research and Development, the Indonesian Ministry of Health (Jakarta, Indonesia), the Ethics Committee of Menzies School of Health Research (Darwin, Australia) and the Oxford Tropical Research Ethics Committee. Informed consent was obtained from all adult participants and from parents of children. An independent Data Safety Monitoring Committee (DSMC) was established prior to commencing the study and was asked to review the progress of the study after 100 patients had been enrolled. The trial was registered with the clinical trials website (http://www.clinicaltrials.gov/ct) as NCT 00157859.

## 3 Results

Between April and September 2004, 143 patients with uncomplicated malaria were enrolled in the study. In total, 103 (72%) had *P. falciparum* infection alone or mixed with *P. vivax* infection and were treated with CQ+SP. The remaining 40 patients (28%) had pure *P. vivax* infections and were treated with CQ monotherapy. Baseline characteristics are given in Table 1.

**Table 1:** Baseline characteristics of uncomplicated malaria study patients

	*P. falciparum* (chloroquine+ sulfadoxine–pyrimethamine)	*P. vivax* (chloroquine)
No. of subjects enrolled	103	40
Males, % (*n*)	61% (63)	55% (22)

Age		
Median (range) (years)	16 (1–60)	14.5 (1.6–60)
<5, % (*n*)	12% (12)	28% (11)
5–14, % (*n*)	35% (36)	23% (9)
>14, % (*n*)	53% (55)	50% (20)

Temperature >37.5 °C, % (*n*)	35% (36)	15% (6)
Splenomegaly, % (*n*)	84% (83/99)	79% (31/39)

Haemoglobin		
Mean (SD) (g/dl)	10.2 (1.9)	10.5 (2.1)
<10 g/dl, % (*n*)	45% (46)	38% (15)

Geometric mean (95% CI) parasite count per μl blood	1851 (1241–2761)	584 (343–993)
Papuan ethnicity, % (*n*)	57% (59)	65% (26)

### 3.1 Tolerability

**Figure 1:**
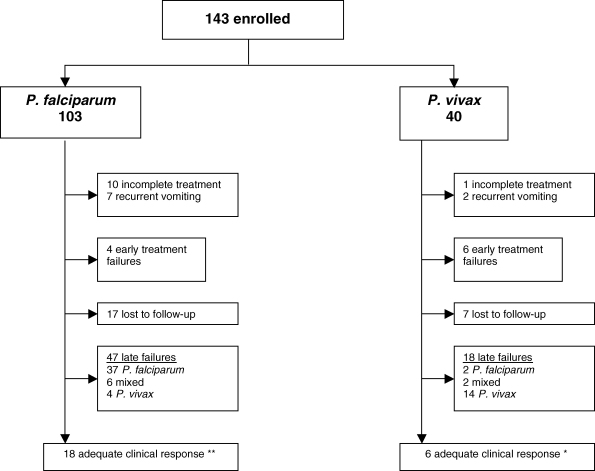
Study profile. ^*^Day 28; ^**^Day 42.

Early vomiting within the first hour of drug administration occurred in 8% of patients (8/103) treated with CQ+SP and 10% of patients (4/40) treated with CQ. Vomiting after CQ administration did not differ between the species of infection, but was 7.2-fold (95% CI 1.7–31) higher in children compared with adults: 19% (13/68) and 3% (2/75), respectively (*P* = 0.003). In total, 6.3% of patients (9/143) were unable to tolerate their medication owing to recurrent vomiting (seven in the CQ+SP group and two in the CQ group), only one of whom was an adult. Five of these patients tolerated changing medication to oral quinine, but two children required i.v. quinine. A further 7.0% of patients (10/143) withdrew consent prior to completion of therapy (four children and six adults). One patient with *P. vivax* infection on admission was treated with CQ but was found to have mixed *P. falciparum* and *P. vivax* infection on Day 1 and treatment was changed to quinine. The study profile is shown in Figure 1.

Of those subjects who received a full treatment course, the mean dose of CQ administered was 27.6 mg/kg (SD 2.9 mg) in the CQ+SP group and 26.3 mg/kg (SD 2.6 mg) in the CQ group. Follow-up to Day 28 or day of failure in these patients was achieved in 90% (77/86) of those treated with CQ+SP and 84% (31/37) following CQ administration.

### 3.2 Early therapeutic response

Early treatment failure (ETF) with CQ+SP occurred in 4.2% of patients (3/71) with *P. falciparum* infection compared with 6.7% of patients (1/15) with mixed infection (*P* = 0.5). Although ETF was more likely in patients with pure *P. vivax* infections treated with CQ (16%; 6/37), the difference did not reach statistical significance (*P* = 0.06). Four patients developed warning signs or markers of severity and required rescue with i.v. quinine. One adult with *P. falciparum* developed convulsions and coma, and one child with falciparum malaria developed respiratory distress on Day 1. An adult and a child, both with vivax malaria, developed severe vomiting and diarrhoea on Day 1. The remaining six patients were categorised as having ETF due to delayed parasite clearance. Early treatment failure was 8.8-fold (95% CI 2.7–28) higher in infants compared with older children and adults (*P* < 0.001).

The median PRR at 48 h was 57 (range <1–1512) for *P. falciparum* and 7.5 (range 1–180) for *P. vivax* (*P* < 0.001)*.* By Day 3, 29% of patients (8/28) infected with pure *P. vivax* had failed to clear their parasitaemia compared with 38% (5/13) of patients with mixed infection and 7% of patients (4/56) with pure *P. falciparum* (*P* = 0.004). However, fever clearance times were generally rapid, with 97% of patients (30/31) having defervesced within 48 h.

### 3.3 Late therapeutic response

During subsequent follow-up, recurrence of malaria was observed in 65 patients, 39 of whom had *P. falciparum*, 18 had *P. vivax* and 8 had mixed infections (see Table 2). Overall, 31% of patients (20/65) with recurrent parasitaemia were symptomatic at the time of treatment failure (reporting malaise, fever or history of fever) and 54% (22/41) were anaemic (haemoglobin <10 g/dl). There were no differences in symptoms observed between the species of infection at the time of failure.

**Table 2:** Failure rates (95% CI) of patients according to initial species of infection

	*P. falciparum*/mixed	*P. vivax*
Early treatment failure	4.7% (1.8–11.4)	16% (7.7–31)
Failure rate by Day 7	13% (5.7–20)^a^	28% (13–43)^b^
Failure rate by Day 28	56% (44–68)^a^	65% (49–81)^b^
Failure rate by Day 42, overall	70% (56–83)^a^	–
Failure rate by Day 42, PCR corrected	48% (31–65)^a^	–
Median time to recrudescence (range) (days)	15 (1–43)	12 (1–22)

a Early treatment failures and reappearance of falciparum or mixed infections.

b Early treatment failures and reappearance of vivax or mixed infections.

**Figure 2:**
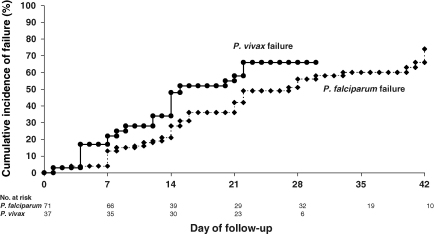
Cumulative incidence of therapeutic failure in patients following treatment of *Plasmodium vivax* with chloroquine (●) and *P. falciparum* with chloroquine plus sulfadoxine–pyrimethamine (♦).

The cumulative Day 28 failure rate for *P. falciparum* was 58% (95% CI 46–69) compared with 74% (95% CI 50–98) for patients with mixed infections and 65% (95% CI 49–81) for pure vivax infections (Table 2). By Day 42, 70% (95% CI 56–83) of patients with either *P. falciparum* or mixed infection had had a recurrence of *P. falciparum* infection. Treatment failure by Day 42 following falciparum malaria occurred in 88% (95% CI 70–100) of children compared with 59% (95% CI 38–80) of adults (hazard ratio = 2.4, 95% CI 1.3–4.3; *P* = 0.004). The same trend was apparent 28 days after treatment of vivax malaria: 82% (95% CI 64–100) of children and 49% (95% CI 24–74) of adults (*P* = 0.004). Survival plots of the cumulative incidence rate of therapeutic failure are presented in Figure 2.

Parasite genotyping was performed in 77% of patients (33/43) with *P. falciparum* or mixed infections on enrolment and at treatment failure. In 17 cases the recurrent isolate was identified as a true recrudescence, in 10 cases as a new infection and in 6 cases the result was indeterminate. The failure rate of *P. falciparum* by Day 42, corrected by PCR and including ETFs, was 48% (95% CI 31–65).

The initial speed of parasite reduction was correlated with subsequent treatment failure in patients infected with *P. vivax* but not with *P. falciparum*. The PRR of *P. vivax* 48 h after starting CQ therapy was 20 (range 4–180) for those vivax successfully treated compared with 7 (range 1–148) in those with recurrence of *P. vivax* by Day 28 (*P* = 0.05). The PRR of *P. vivax* at 24 h was significantly correlated with the day of recurrence (Pearson's coefficient = 0.53, *P* = 0.018). Using Youden's Index, the best cut-off at 24 h was a PRR of 7.4; parasite reduction below this level predicted subsequent treatment failure with 70% sensitivity and 63% specificity.

### 3.4 Chloroquine concentrations

On admission, 39% of patients (35/89) had measurable concentrations of plasma CQ (see Table 3). In 29% of patients (18/63) infected with *P. falciparum* or mixed infections and in 27% of patients (7/26) presenting with vivax malaria, plasma CQ concentrations were above the MEC. The plasma CQ concentrations on Day 7 were significantly lower in children (median 92 ng/ml, range 0–426 ng/ml) compared with adults (median 232 ng/ml, range 0–578 ng/ml) (*P* = 0.0001) but did not differ by species of infection. The difference was still apparent after excluding those with early vomiting. Three patients (one adult and two children) had undetectable CQ concentrations on Day 7; all of these patients failed therapy within 21 days.

**Table 3:** Plasma chloroquine levels

	*P. falciparum*	*P. vivax*
Day 0		
Detectable levels, % (*n*)	44% (28/63)	27% (7/26)
Median concentration (range) (ng/ml)^a^	52.5 (9–215)	97 (27–208)

Day 7		
Detectable levels, % (*n*)	94% (33/35)	91% (10/11)
Median concentration (range) (ng/ml)	181 (0–578)	168 (0–426)

Day of failure		
Detectable levels, % (*n*)	78% (18/23)	100% (7/7)
Median concentration (range) (ng/ml)	44 (0–242)	44 (25–108)

a In those with detectable levels.

Plasma samples were available for CQ analysis in 49% of patients (23/47) who recrudesced with *P. falciparum* or mixed infections. In these patients, the median concentration of CQ+DCQ was 43 ng/ml (range 0–242 ng/ml), exceeding the MEC in 61% of cases (14/23). In patients with *P. vivax*, CQ concentrations were measured in 7 (39%) of 18 patients during follow-up. The median CQ+DCQ concentration on the day of recrudescence was 44 ng/ml (range 25–108 ng/ml), with all of these patients having concentrations in excess of the MEC.

### 3.5 Gametocyte carriage following treatment

On admission, gametocytes were present in 9% of patients (8/87) infected with *P. falciparum* compared with 18% (7/40) with *P. vivax* and 38% (5/13) with mixed infections (*P* = 0.004). By Day 7, none of the patients with *P. vivax* infections had gametocytes present on blood film examination compared with 47% (32/68) following treatment of *P. falciparum* and mixed infections (*P* < 0.001). Following *P. falciparum* infection, gametocyte carriage was still present in 29% of patients (7/24) on Day 28. At the time of recrudescence, gametocytes were present in 34% of patients (22/64) failing therapy, with no difference between species of infection.

### 3.6 Re-treatments

Overall, 49 patients with *P. falciparum* or mixed infections had treatment failures (2 delayed parasite clearance and 47 with late recurrence). Four patients refused re-treatment. A child aged 5 years who had a reappearance of *P. falciparum* that was associated with vomiting and tachypnoea on Day 12 was treated with i.v. quinine as an inpatient. In total, 44 patients agreed to be re-treated with 7 days of unsupervised quinine (28 adults also received doxycycline). There were no ETFs but 67% of patients (22/33) successfully followed-up had a reappearance of parasitaemia by Day 28 (14 patients had *P. falciparum*, 5 patients had mixed infection and 3 patients had *P. vivax* infection). By Day 28 the uncorrected failure rate of unsupervised quinine ± doxycycline for *P. falciparum* was 48% (95% CI 31–65) in this cohort. There was no significant difference in those receiving monotherapy or additional doxycycline.

Twenty-two patients had *P. vivax* treatment failures (4 delayed parasite clearance and 18 with late recurrences). A 16-year-old male re-presented on Day 22 with a recurrence of *P. vivax* associated with tachypnoea and a 3-day history of vomiting everything; he was treated as an inpatient. One adult refused re-enrolment. In total, 20 patients (15 children and 5 adults) agreed to re-treatment and follow-up. Nine patients were re-treated with 7 days of unsupervised quinine, of whom six were successfully followed-up: five had a further reappearance of *P. vivax* and one had an adequate parasitological response. Eleven patients were re-treated with amodiaquine (30 mg base/kg) over 3 days. There were no ETFs. Three were successfully followed to Day 28: two had an adequate parasitological response and one child had a further reappearance of *P. vivax* on Day 31.

## 4 Discussion

This study highlights that the antimalarial policy being used in 2004 in southern Papua, Indonesia, was no longer effective. Almost 5% of patients with *P. falciparum* or mixed infections treated with CQ+SP and 16% of those infected with *P. vivax* treated with CQ alone required early rescue therapy. The failure rate for *P. vivax* had risen to 65% by Day 28 and that for *P. falciparum* or mixed infections had risen to 71% by Day 42. When this degree of resistance became apparent, the study was referred to an independent DSMC who felt that it was inappropriate to continue using these treatment regimens and the study was stopped. These failure rates were based on a per-protocol analysis to define resistance levels of the parasite. In practice a further 6% of patients were intolerant of CQ and another 8% failed to complete therapy. If one assumes that all these patients would have had a recurrence of malaria during the follow-up period, then the overall Day 42 cure rate during the study period would have been nearer 32% for *P. falciparum* and mixed infections and 24% for *P. vivax*. These levels of drug resistance are undermining the local malaria control strategy and contributing a considerable burden of mortality and morbidity upon the local communities.

Efficacy was significantly lower in infants, who were 8.8 times (95% CI 2.7–28) more likely to suffer an ETF. Older children also had an increase risk of late treatment failure of *P. falciparum* 2.4-fold (95% CI 1.3–4.3) higher compared with adults. This may be attributed in part to children being less tolerant of CQ therapy as demonstrated by 7.2-fold (95% CI 1.7–31) higher rates of early vomiting and 60% lower plasma CQ levels on Day 7. However, as in other endemic areas, the lower failure rates in adults are also likely to represent a degree of immunity helping to clear resistant blood-stage parasites from the bloodstream (Price et al., 1998).

Previous studies in Papua New Guinea and Papua Indonesia have shown the coexistence of CQ-resistant strains of *P. falciparum* and *P. vivax*, but until recently the degree of resistance of *P. vivax* has tended to lag behind that of *P. falciparum*. Our data from southern Papua show CQ resistance in *P. vivax* was significantly worse than that of *P. falciparum*. The 48-h PRR of 7.5 for *P. vivax* was significantly lower than the 57 observed for *P. falciparum*. These figures compare with 438 for CQ-sensitive *P. vivax* isolates from Thailand (Pukrittayakamee et al., 2000a) and 1000 for CQ-sensitive *P. falciparum* isolates (White et al., 1989). The *P. vivax* PRR at 24 h was correlated significantly with the day of recurrence, hence the lower the early reduction in parasite biomass the earlier the recrudescence of *P. vivax*. At 24 h, a PRR <7.4 predicted subsequent treatment failure with 70% sensitivity and 63% specificity. This early marker of therapeutic efficacy could have useful application in other areas endemic for *P. vivax* as a rapid assessment for CQ efficacy.

Conducting an in vivo study in an endemic area is confounded by re-infection. However, in this study PCR genotyping confirmed that 63% of the *P. falciparum* treatment failures were due to true recrudescence rather than re-infection and the Day 42 corrected cure rate following *P. falciparum* or mixed infection was 52%. Determining in vivo efficacy of *P. vivax* is more difficult since recurrence of malaria can be due to recrudescence from the same isolate, re-infection with a new isolate or relapse from hypnozoite stages (White, 2002). A review of published records of early clinical trials demonstrated that CQ levels after a dose of 25 mg/kg exceed the MEC of sensitive strains of *P. vivax* for at least 28 days (Baird et al., 1997b). In this region, the first relapse is observed 17–21 days after therapy, with a further relapse at approximately 42 days. Hence, any recurrence of *P. vivax* within 28 days either represents recrudescence or a breakthrough relapse of a drug-resistant isolate (Baird et al., 1997b). In our study, the plasma concentrations of CQ at Day 7 were high and all late reappearances of *P. vivax* with concomitant CQ levels occurred at concentrations of CQ above the recognised MEC (15 ng/ml). This adds further evidence that the failure rates observed in our study represent true CQ-resistant *P. vivax* rather than poor drug absorption.

Although primaquine is ineffective against the asexual stages of *P. falciparum*, it is effective against all stages of *P. vivax* and in combination with CQ may improve efficacy against blood stages of *P. vivax* (Baird et al., 1995). In our study, the high level of CQ resistance against *P. vivax* was not anticipated and terminal eradication was delayed until Day 28. It is possible that the addition of primaquine on admission might have facilitated early parasite clearance and prevented some of the ETFs. However, the efficacy of primaquine against liver stages generally requires 7–14 days of therapy (1 mg/kg or 0.5 mg/kg daily, respectively), a regimen to which adherence is poor in practice. Furthermore, in Papua it is unlikely that co-administration of an unsupervised 14-day course of primaquine would have prevented relapse of resistant isolates at the tail end of CQ treatment because of poor compliance with the lengthy primaquine regimen (Baird and Rieckmann, 2003).

In 2004, second-line therapy for uncomplicated malaria in Mimika district was a 7-day course of unsupervised quinine (plus doxycycline if older than 8 years). In our study, the numbers completing a 28-day follow-up following re-treatment were small but highlighted the 48% failure rate for *P. falciparum* and 70% for *P. vivax*. In Thailand where multidrug-resistant *P. falciparum* is highly prevalent, unsupervised quinine therapy was associated with high failure rates, but cure rates rose to 85% by Day 28 when the drug was supervised (Pukrittayakamee et al., 2000b). Quinine is poorly tolerated, reliably causing cinchonism, and needs to be administered three times a day for 7 days. Hence, although the poor efficacy of this regimen might represent emergence of quinine-resistant isolates, a more likely explanation is treatment failure secondary to poor adherence as well as the inability of quinine, a rapidly eliminated antimalarial, to prevent the first relapse of *P. vivax* (Pukrittayakamee et al., 2000a). In total, 11 patients with recrudescent *P. vivax* were retreated with amodiaquine monotherapy. Early parasite clearance was rapid in all cases with no ETFs. Although only three patients were followed for 28 days, none failed therapy during this time. This is one of the first reports of the efficacy of amodiaquine against CQ-resistant strains. Although it suggests that its efficacy may be superior to CQ, further studies are needed to confirm this.

In summary, our findings highlight the high levels of treatment failure both of first- and second-line therapies for *P. falciparum* and *P. vivax* in southern Papua. The current level of resistance is likely to be the most important factor in the inability of the established malaria control programme to decrease the large burden of disease. Alternative strategies involving the use of artemisinin-based combination therapies are in progress.

## Conflicts of interest statement

The authors have no conflicts of interest concerning the work reported in this paper.
